# Identifying the predictors of ultra early neurological improvement and its role in functional outcome after endovascular thrombectomy in acute ischemic stroke

**DOI:** 10.3389/fneur.2025.1492013

**Published:** 2025-01-31

**Authors:** Yuzheng Lai, Mohammad Mofatteh, José Fidel Baizabal-Carvallo, Jianfeng He, Wenhao Wu, Daohong Wang, Wenshan Yan, Jicai Ma, Sijie Zhou, Yu Sun, Yi He, Shumei Li, Hao Sun

**Affiliations:** ^1^Department of Neurology, Guangdong Provincial Hospital of Integrated Traditional Chinese and Western Medicine (Nanhai District Hospital of Traditional Chinese Medicine of Foshan City), Foshan, China; ^2^School of Medicine, Dentistry and Biomedical Sciences, Queen’s University Belfast, Belfast, United Kingdom; ^3^Parkinson's Disease Center and Movement Disorders Clinic, Department of Neurology, Baylor College of Medicine, Houston, TX, United States; ^4^Department of Sciences and Engineering, University of Guanajuato, León, Mexico; ^5^The Second Clinical Medical College, Guangdong Medical University, Zhanjiang, China; ^6^Department of Neurology, The Affiliated Yuebei People’s Hospital of Shantou University Medical College, Shaoguan, China; ^7^Department of Surgery of Cerebrovascular Diseases, First People's Hospital of Foshan, Foshan, China; ^8^Department of Neurology, Xiapu County Hospital, Ningde, China; ^9^Department of Neurology and Advanced National Stroke Center, Foshan Sanshui District People's Hospital, Foshan, China; ^10^Intervention Center, Guangdong Provincial Hospital of Integrated Traditional Chinese and Western Medicine (Nanhai District Hospital of Traditional Chinese Medicine of Foshan City), Foshan, China

**Keywords:** endovascular therapy, thrombectomy, acute ischemic stroke, anterior circulation, patient outcome, neurological improvement, NIHSS

## Abstract

**Background and purpose:**

Using post-treatment methods to predict functional outcomes of acute ischemic stroke (AIS) patients undergoing endovascular thrombectomy (EVT) is crucial in stroke medicine. The National Institute of Health Stroke Scale (NIHSS) score at 24 h has been widely used; however, there is a paucity of data on using earlier NIHSS scores and their association with outcome. In this study, we aimed to investigate the usage of NIHSS at 1-h time window -ultra-early neurological improvement (UENI)- as a surrogate marker associated with the functional outcomes of AIS patients treated with EVT.

**Methods:**

We included 485 adults (≥18 years old) who underwent emergency EVT at four academic comprehensive stroke centers between 2020 and 2021. Patients with pre-EVT Alberta Stroke Program Early CT Score (ASPECTS) < 6, missing follow-up data, and missing data of the first hour NIHSS were excluded (*n* = 20). UENI was defined as post-EVT NIHSS reduction of 4 points or more or NIHSS as 0–1 within 1-h post-EVT. An mRS score of 0–2 after three months was defined as favorable outcome, and independent walking independence was defined as mRS of 3.

**Results:**

A total of 465 patients were included in our final analysis. We identified 122 (26.2%) patients with UENI. While 82.79% of the patients with UENI achieved favorable functional outcomes at 3-months, only 32.36% of patients without UENI had favorable functional outcome (*p* < 0.0001). In addition, lower hospitalization costs were associated with patients who had UENI, compared to No-UENI (*p* = 0.003). A multivariate logistic regression analysis revealed that younger age (*p* < 0.0001), shorter last know normal to puncture time (LKNPT) (*p* = 0.013), higher pre-treatment ASPECTS (*p* = 0.039), final modified thrombolysis in cerebral infarction (mTICI) ≥2b (*p* = 0.002), and fewer number of EVT attempts (*p* = 0.002) were variables independently associated with UENI. The presence of UENI was independently associated with a better outcome OR: 7.999 (95% C.I. 4.415–14.495).

**Conclusion:**

UENI was observed in about a quarter of patients with AIS undergoing EVT. Younger age, shorter LKNPT, higher pre-treatment ASPECTS, final mTICI≥2b, and fewer number of EVT attempts, were independently associated with UENI. The presence of UENI was independently associated with better functional outcome at 3 months.

## 1 Introduction

Despite recent advances in providing neurological care, stroke remains one of the major causes of mortality and patient disability worldwide (1). Endovascular thrombectomy (EVT) has significantly improved the treatment of acute ischemic stroke (AIS) and has become the standard of care in many neurological and critical care centers globally (2, 3).

As it has been reported in multiple studies, early neurological improvements can be used as a surrogate marker for predicting the functional outcome of AIS patients undergoing EVT (4–10). For instance, a surrogate for long-term outcome after EVT can be a reduction in the National Institute of Health Stroke Scale (NIHSS) score of larger than 4 points or NIHSS of 0 or 1 at 24 h (11). Therefore, identification of surrogates for early neurological improvement can be crucial in predicting longer-term outcomes of AIS patients with large vessel occlusion undergoing EVT.

In the current study, we aimed to evaluate the predictors of Ultra Early Neurological Improvement (UENI) after EVT and the relationship between UENI and outcome. We defined the Ultra Early Neurological Improvement as post-EVT NIHSS reduced by 4 points or more or NIHSS as 0–1 within 1 h after EVT (12).

## 2 Methods

### 2.1 Study design

We conducted a retrospective analysis of prospectively collected data from patients who underwent EVT at four academic comprehensive stroke centers in China from 2020 to 2021. The patients’ data was derived from the Big Data Observatory Platform for stroke in China and from the hospital data platform.

Inclusion criteria were as follows: (1) patients who underwent emergency EVT; (2) age ≥ 18 years old; (3) within 24 h from onset. Exclusion criteria were as follows: (1) pre-EVT Alberta Stroke Program Early CT Score (ASPECTS) < 6 (lower scores denotes greater parenchymal involvement), (2) missing follow-up data and, (3) missing NIHSS data within the first hour following EVT.

### 2.2 Data collection

We collected the baseline patient characteristics, risk factors of cerebrovascular disease, initial premorbid modified Rankin Scale (mRS), door-to-needle time (DNT), onset-to-needle time (ONT), door-to-puncture time (DPT), last know normal-to-puncture time (LKNPT), door-to-recanalization time (DRT), modified thrombolysis in cerebral infarction (mTICI) post thrombectomy. Successful reperfusion was defined as mTICI ≥2b. At least two attending neurologists collected data together. In case of discrepancies in data collection or interpretation, a consensus by the group was reached under the supervision of a senior clinician. As per the requirements by the China Stroke Center protocol, all EVT patients should have their NIHSS assessed within 1 h after EVT. The NIHSS score was assessed prospectively.

### 2.3 Outcome measures

The patients’ outcomes were evaluated by the mRS at 3 months after EVT. Favorable outcome was defined as mRS of 0–2. Walking independence was defined as mRS of ≤3.

### 2.4 Statistical analysis

The non-parametric Mann–Whitney U test was performed using the IBM SPSS 26 version (IBM-Armonk, NY) to analyze non-normally distributed continuous data, reported as medians along with the interquartile range (IQR). Normally distributed data are reported as means with corresponding standard deviations (SD) and compared using the student’s t-test. We carried out a multivariate logistic regression analysis with the backward Wald’s method and presence of “UENI” as the dependent variable to assess the effect of variables showing a statistically significant association in the bivariate analysis. The exp. (B) coefficient as odds ratios with 95% confidence interval (C.I.) were used to standardize the weight of independent variables and assess their association with the presence of UENI. The Hosmer-Lemeshow test was used to assess goodness of fit and calibration of the regression model and the Nagelkerke test to calculate the determination coefficient R^2^ of the model. The same procedure was used to assess the effect of UENI as independent variable in the 3-months outcome with “favorable outcome (mRS 0–2)” as the dependent variable. For the latter model, we included only variables independently associated with a favorable outcome at 3 months. Results were considered statistically significant if the *p*-value was less than 0.05.

### 2.5 Ethics approval and consent to participate

The study protocol was approved by the hospital’s institutional review board. Informed consents were waived due to the retrospective nature of the study in compliance with national laws and regulations. All procedures performed in the studies involving human participants were in accordance with the ethical standards of the institutional and/or national research committee and with the 1964 Declaration of Helsinki and its later amendments or comparable ethical standards.

## 3 Results

There were 485 patients initially, four of whom were excluded due to loss at follow-up, four patients were excluded due to pre-treatment ASPECTS score < 6, and 12 patients were excluded due to missing NIHSS data within the first hour following EVT. After the application of the inclusion and the exclusion criteria, 465 patients were enrolled in our study. Patients were divided into two groups: No-UENI (*n* = 343, 73.8%) and UENI (*n* = 122, 26.2%). There were statistically significant differences between age (*p* = 0.001), pre-treatment ASPECTS (*p* = 0.0001), vessel occlusion site (*p* = 0.030), DRT (*p* = 0.025), LKNPT (*p* = 0.002), mTICI of 3 (*p* = 0.027), number of EVT passes (*p* = 0.001), and symptomatic intracranial hemorrhage (sICH) (*p* = 0.003) between groups (Table 1). Internal carotid artery occlusion (isolated or in tandem with middle cerebral artery) were less common in patients with UENI: 35 (28.68%) vs. 135 (39.35%), (*p* = 0.036). In summary, patients with UENI were younger had higher pre-treatment APECTS scores, shorter DRT and LKNPT, required a lesser number of EVT passes and achieved more frequently mTICI ≥2b or 3. They also had less frequently tandem ICA + MCA-M1 occlusions and sICH. Sex and risk factors for ischemic stroke did not differ between groups.

**Table 1:** Comparison of baseline characteristics UENI and No-UENI.

	No-UENI	UENI	*X*^2^/t/z	*p*
Number	343	122		
Age mean ± SD	66.29 ± 11.95	62.03 ± 12.23	3.358	0.001**
Male sex, *n*, %	229 (66.76)	91 (74.59)	2.569	0.109
Stroke risk factors				
Hypertension, *n*, %	222 (64.72)	72 (59.02)	1.260	0.262
Diabetes mellitus, *n*, %	76 (22.16)	22 (18.03)	0.920	0.337
CAD, *n*, %	64 (18.66)	23 (18.85)	0.002	0.962
Atrial fibrillation, *n*, %	118 (34.40)	42 (34.43)	0.000	0.996
Prior stroke, *n*, %	71 (20.70)	23 (18.85)	0.190	0.663
Hyperlipidemia, *n*, %	79 (23.03)	30 (24.59)	0.122	0.727
Smoker, *n*, %	87 (25.36)	37 (30.33)	1.134	0.287
Pre-EVT NIHSS (IQR)	16.000 (12.0,21.0)	16.000 (12.0,19.3)	−1.042	0.298
pre-treatment ASPECTS	8.06 ± 1.12	8.50 ± 1.00	−3.816	0.000**
mRS premorbid (IQR)	0.000 (0.0,0.0)	0.000 (0.0,0.0)	−1.026	0.305
Vessel occlusion site				
ICA *n*, %	53 (15.45)	15 (12.30)	12.366	0.030*
MCA-M1 *n*, %	114 (33.24)	61 (50.00)		
MCA-M2 *n*, %	29 (8.45)	6 (4.92)		
Tandem *n*, %	82 (23.91)	20 (16.39)		
Basilar	41 (11.95)	15 (12.30)		
Others	24 (7.00)	5 (4.10)		
ICA total *n*, %	135 (39.35)	35 (28.68)	4.417	0.036*
Intravenous thrombolysis, *n*, %	121 (35.28)	50 (40.98)	1.260	0.262
DPT (IQR), min	122.000 (75.0,175.0)	107.000 (77.8,153.0)	−1.148	0.251
DRT (IQR), min	188.000 (125.0,260.0)	158.000 (115.0,237.0)	−2.239	0.025*
LKNPT (IQR), min	320.000 (205.0,590.0)	257.000 (164.0,450.0)	−3.101	0.002*
mTICI 2b-3	286 (83.38)	121 (99.18)	20.574	0.000
mTICI of 3	206 (60.06)	87 (71.31)	4.889	0.027*
Number of EVT passes	1.79 ± 1.18	1.44 ± 0.87	3.423	0.001**
sICH, *n*, %	35 (10.20)	2 (1.64)	9.013	0.003**

ASPECTS, Alberta Stroke Program Early CT Score; CAD, coronary artery disease; DPT, door-to-puncture time; DRT, door-to-recanalization time; EVT, endovascular thrombectomy; ICA, internal carotid artery; IQR, interquartile range; LKNPT, last-known normal-to-puncture time; MCA, middle cerebral artery; mRS, modified Rankin scale; mTICI, modified thrombolysis in cerebral infarction; NIHSS, National Institute of Health Stroke Scale; sICH, symptomatic intracranial hemorrhage; UENI, ultra-early neurological improvement. *Denotes significance.

After the multivariate regression analysis, younger age, large vessel occlusion site, shorter LKNPT, higher pre-treatment ASPECTS, mTICI final≥2b, and fewer EVT attempts were independently associated with UENI (Table 2). The 90-day mRS scores distribution is shown in Supplementary Table 1.

**Table 2:** Analysis of regression of factors associated with UENI.

Variables	Coefficient	S.E.	Wald *χ*^2^	*p*	OR	OR 95% CI
Age	−0.038	0.010	15.006	0.000	0.963	0.945 ~ 0.982
LKNPT	−0.001	0.000	9.355	0.002	0.999	0.998 ~ 0.999
pre-treatment ASPECTS	0.245	0.112	4.774	0.029	1.278	1.026 ~ 1.593
Final mTICI ≥ 2b	3.224	1.033	9.738	0.002	25.123	3.317 ~ 190.295
Number of EVT attempts	−0.381	0.124	9.375	0.002	0.683	0.535 ~ 0.872

Constant: −2.522, Hosmer-Lemeshow: *χ*^2^ = 7.518, *p* = 0.482. Nagelkerke *R*^2^ = 0.212. EVT, endovascular thrombectomy; LKNPT, last-known normal-to-puncture time. Variables not included in the final model: internal carotid artery occlusion and sICH, symptomatic intracranial hemorrhage.

**Figure 1:**
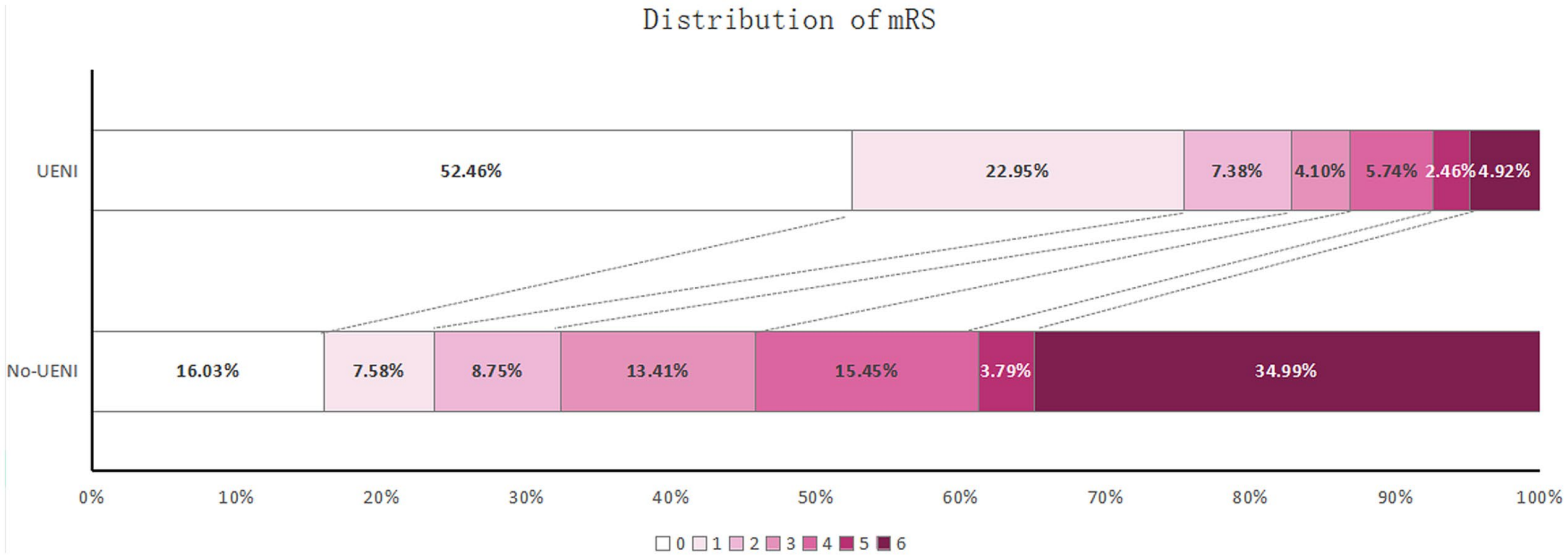
Distribution of 3-months mRS scores in No-UENI and UENI patients.

Our analysis demonstrated that 82.79% of patients achieved favorable outcomes in the UENI group, while only 32.36% of patients achieved favorable outcomes in the No-UENI group. In addition, the UENI group had fewer hospitalization costs compared with the No-UENI group (median 84171.150 RMB vs. 91244.665 RMB) (Table 3). The mRS scores distribution between groups after 3 months is shown in Figure 1. In the multivariate regression analysis, age, diabetes mellitus, NIHSS score at presentation, pre-treatment APECTS, intravenous thrombolysis, final mTICI ≥2band sICH were included in the final model, along with UENI (OR: 7.999, 95% C.I. 4.415–14.495) (Table 4). Suggesting that UENI is independently associated with a better 3-month prognosis in stroke patients undergoing thrombectomy.

**Table 3:** Comparison of outcome of UENI and No-UENI.

	No-UENI	UENI	*X*^2^/z	*p*
Hospitalization costs (RMB)	91244.665 (72805.9, 123752.8)	84171.150 (59991.8, 104464.9)	−2.941	0.003*
90-day favorable outcome, n, %	111 (32.36)	101 (82.79)	92.246	0.000*

sICH, symptomatic intracranial hemorrhage; UENI, ultra-early neurological improvement. Favorable outcome: mRS (0–2) at 3-months. Poor outcome: mRS ≥ 3. Hospitalization cost was calculated in Chinese Yuan (RBM). * Denotes significance.

**Table 4:** Analysis of regression of factors associated with favorable outcome (mRS 0–2) at 3 months.

Variables	Coefficient	S.E.	Wald *χ*^2^	*p*	OR	OR 95% CI
Age	−0.023	0.010	5.395	0.020	0.977	0.958 ~ 0.996
Diabetes mellitus	−0.654	0.284	5.290	0.021	0.520	0.298 ~ 0.908
NIHSS score at presentation	−0.096	0.020	23.888	0.000	0.909	0.874 ~ 0.944
pre-treatment ASPECTS	0.276	0.114	5.857	0.016	1.317	1.054 ~ 1.647
Intravenous thrombolysis	−0.414	0.256	2.621	0.105	0.661	0.400 ~ 1.091
UENI	2.079	0.303	47.007	0.000	7.999	4.415 ~ 14.495
Final mTICI ≥ 2b	1.424	0.475	8.977	0.003	4.156	1.637 ~ 10.552
sICH	−20.723	6096.4	0.000	0.997	0.000	0.000

Constant: −0.728, Hosmer-Lemeshow: *χ*^2^ = 10.559, *p* = 0.228. Nagelkerke *R*^2^ = 0.459. Variables not included in the final model: last know normal time to puncture, number of EVT passes, large vessel occlusion artery. NIHSS, National Institute of Health Stroke Scale; sICH, symptomatic intracranial hemorrhage; UENI, ultra-early neurological improvement.

## 4 Discussion

In this multi-center retrospective study of 465 AIS patients, we aimed to evaluate the NIHSS score shortly following the EVT procedure in order to define a group of patients with UENI and assessed if this variable associated with a better functional outcome of AIS at follow-up. In our study, we defined UENI as a reduction of 4 or more points in post-EVT NIHSS or NIHSS as 0–1 within 1 h following EVT. We found that 26.2% of patients fulfilled the criteria. No differences in sex distribution or stroke risk factors were observed between patients with UENI and No-UENI. Moreover, no differences in the functional status (mRS at presentation) before the AIS event were observed between these groups.

Our data revealed that in the UENI group, 82.79% of patients achieved favorable outcomes, whereas favorable outcomes were only achieved in 32.36% of patients in the No-UENI group. In addition, there was less hospitalization costs associated with patients who had UENI (*p* = 0.003). Furthermore, UENI patients achieved favorable outcomes more frequently at 3 months, compared with the No-UENI group (*p* < 0.0011).

Making appropriate medical and surgical decisions in the acute phase of AIS is important, as patients’ outcomes often rely on the usage of medium and/or long-term prognosis indicators (13). Baseline factors and scores, such as NIHSS have been routinely used as outcome predictors for patients with AIS (14).

It is important to acknowledge that since the advent of EVT, post-treatment measures have gained significance and have become as important as baseline measures for predicting functional outcomes in patients with AIS (14). Therefore, it is reasonable to hypothesize that post-EVT NIHSS score can be used as an appropriate surrogate for the assessment of AIS patient prognosis (15–19).

There have been slight disagreements between different studies regarding the exact definition of early neurological improvements. However, it has been generally defined as 24-h NIHSS improvement of ≥8 or 10 points or a 24-h NIHSS score of 0–1 (2, 18, 19). In addition, other studies reported that early trajectory of the post-EVT NIHSS score within two days can be an accurate predictor of functional outcome (14). However, the significance of ultra-early post-EVT indicators, namely post-EVT NIHSS, has remained understudied (20).

A major advantage of using an NIHSS score shortly after EVT for functional outcomes is that it summarizes the effect of other variables with potential prognostic weight into a single score in order to predict the functional outcome few months later, providing a potential frame to guide clinical and surgical decisions (20). In our study, younger age at AIS presentation, less parenchymal involvement assessed with ASPECTS, shorter ischemic time determined with LKNPT, greater recanalization flow by the mTICI score and less thrombotic-occlusion burden reflected by the lower number of EVT passes, related to a UENI.

Different studies have reported more discriminative power in predicting desirable functional outcomes could be achieved by incorporating immediate post-EVT NIHSS (21). In addition, constructing several multivariable regression analysis models revealed that those models which incorporated post-EVT NIHSS were more successful in predicting the patients’ outcomes (21). Similarly, another study revealed that functional outcomes can be predicted with high accuracy using early trajectory of NIHSS score within 48 h post-EVT (14). Our study is subject to some limitations. This is a retrospective study of prospectively collected data with a relatively small sample size, which can compromise the external validity of the findings. As a retrospective study, patient data collection and recording could be at risk of recall and classification bias, despite all the efforts for standardization between all 4 centers involved in the study. Patients with an ASPECTS score below 6 were excluded. This exclusion criterion removed cases with greater parenchymal involvement, which may limit the generalizability of the results to patients with more severe AIS. Future studies with more inclusive criteria are required to generalize these findings. It is also important to consider that the current study was conducted in China, which has a significant stroke burden. Findings from this study can differ from results observed in different healthcare settings using different stroke care protocols or covering patients with different demographics. Future large-scale multi-center international studies are required to address these limitations. Despite such limitations, disseminating these findings from the current study can prompt other stroke centers across the globe to use UENI to predict functional patient outcomes. In addition, findings from the current study can be used in combination with other factors to build predictive models for the prognosis of AIS patients undergoing EVT (22–29).

## 5 Conclusion

Appropriate medical and surgical decisions in the acute phase of AIS rely on the usage of medium and/or long-term indicators of prognosis. We showed that younger age, higher pre-treatment ASPECTS, shorter LKNPT, mTICI 2b-3 and, fewer numbers of EVT attempts were associated with UENI. In addition, UENI related to fewer hospitalization costs and more frequent favorable outcomes at 3 months. Data obtained from our study underscores that UENI can be associated to functional outcome, but further larger studies should confirm UENI as a reliable independent surrogate of favorable functional outcome in patients with AIS undergoing EVT.

## Data Availability

The raw data supporting the conclusions of this article will be made available by the authors, without undue reservation.
