# Supplementary material for: Identifying the predictors of ultra early neurological improvement and its role in functional outcome after endovascular thrombectomy in acute ischemic stroke
Source: Front Neurol. 2025 Jan 31;16:1492013. doi: 10.3389/fneur.2025.1492013 (PMC11825449; doi:10.3389/fneur.2025.1492013)
Supplement: Supplementary file 1 [file Table_1.docx]

Supplementary Material

# Supplementary Table 1. 90-day mRS

|  | **No-UENI** | **UENI** | *X^2^/t/z* | *p* |
| --- | --- | --- | --- | --- |
| 90-day mRS of 0 | 55 (16.03) | 64 (52.46) | 109.348 | 0.000** |
| 90-day mRS of 1 | 26 (7.58) | 28 (22.95) |  |  |
| 90-day mRS of 2 | 30 (8.75) | 9(7.38) |  |  |
| 90-day mRS of 3 | 46 (13.41) | 5 (4.10) |  |  |
| 90-day mRS of 4 | 53 (15.45) | 7 (5.74) |  |  |
| 90-day mRS of 5 | 13 (3.79) | 3 (2.46) |  |  |
| 90-day mRS of 6 | 120 (34.99) | 6 (4.92) |  |  |

mRS, modified Rankin scale; UENI, ultra-early neurological improvement.
